# World Water Day — March 22, 2013

**Published:** 2013-03-22

World Water Day, sponsored by the United Nations (UN), is observed every year on March 22. This year, World Water Day focuses on water cooperation.

Since 1990, the number of persons able to access improved drinking water and sanitation resources has increased by nearly 2 billion and 1.8 billion, respectively (1). Despite these gains, hundreds of millions still lack access to these essential resources (1).

In December 2010, the UN General Assembly declared 2013 as the International Year of Water Cooperation. This observance aims to promote water cooperation across different types of organizations and governments and across different disciplines. Water cooperation is a foundation for peace and sustainable development because 1) it is key to poverty eradication, social equity, and gender equality; 2) it creates economic benefits from more efficient and sustainable uses of water resources; 3) it is crucial to preserving water resources and protecting the environment; and 4) it builds peace through partnerships on such a practical and vital issue.

Additional information about World Water Day and ideas on how to get involved are available via the UN World Water Day website at http://www.unwater.org/worldwaterday. Information on CDC’s efforts to ensure global access to improved water, sanitation, and hygiene resources is available at http://www.cdc.gov/healthywater/global.
